# Digitalization in Urology—A Multimethod Study of the Relationships between Physicians’ Technostress, Burnout, Work Engagement and Job Satisfaction

**DOI:** 10.3390/healthcare11162255

**Published:** 2023-08-10

**Authors:** Clara Bail, Volker Harth, Stefanie Mache

**Affiliations:** Institute for Occupational and Maritime Medicine (ZfAM), University Medical Center Hamburg-Eppendorf (UKE), 20459 Hamburg, Germany; clara.bail@stud.uke.uni-hamburg.de (C.B.); harth@uke.de (V.H.)

**Keywords:** technostress, burnout, work engagement, job satisfaction, urology, ICT, digitalization, Germany, multimethod

## Abstract

The potential influences of digitization on the mental health of personnel in the healthcare sector are increasingly coming into the scientific focus in the healthcare sector, especially in terms of the use of information and communication technologies. To date, there have been no German studies of the effects of technostress in healthcare. This cross-sectional study examined the relationships between technostress, burnout, work engagement, and job satisfaction among physicians in the field of urology. Data were collected via an online survey based on the job demands–resources model and the concept of technostress. The survey was sent to German urologists working in inpatient clinics. The participating physicians experienced moderate levels of technostress (M = 2.67, SD = 0.69). The results, based on a general linear model analysis, showed that technostress is significantly positively associated with burnout (β = 0.293; *p* < 0.001) and negatively associated with work engagement (β = −0.175; *p* < 0.001) and job satisfaction (β = −0.206; *p* < 0.001). This study also identified stress and strain factors associated with the use of ICT and assessed institutional support offers as coping mechanisms. The results of this study and its formulated practical implications can serve as a basis for discussing sustainable digitalization strategies in hospitals, taking into consideration technostress and its impact on physicians’ burnout, work engagement and job satisfaction.

## 1. Background

The transformation to a digitalized working world poses unforeseen and new challenges in relation to the implementation of new technologies [1]. These technologies include PCs, mobile devices, the internet, artificial intelligence, autonomous systems, wearables, and augmented and virtual reality. The acceleration and intensification of work processes and relationships that occur as a result of these new technologies are creating major challenges for organizations and their employees. As these technologies become more prevalent in the health sector, more light is shed on the possible influences of digitalization on mental health, especially in terms of the use of information and communication technologies (ICT) and the resulting mental stress and strain factors [2].

## 2. Introduction

### 2.1. Digitalization in Medicine

Digital information and communication technologies are developing rapidly in the health sector and are being used more and more frequently [3]. This includes the digitalization of numerous health services [4] and practices, the development of mHealth, health information technology, wearables, telemedicine, health portals and personalized medicine [5]. As digitalization can improve the quality of patient care, reduce costs, and increase the overall cost efficiency in healthcare [6], this market is expected to grow by almost 50% in Germany by 2025 compared to 2019, not least due to being accelerated by the COVID-19 pandemic [7].

In urology, newer technologies have been rapidly embraced in the past and, in many cases, improved upon to achieve better patient outcomes [8]. A recent survey of German urologists working in outpatient and inpatient care showed that 70% of the respondents already use digital medical records, 38% participate in web-based tumor conferences, and 12% use video consultations. Moreover, 78% consider the digital transformation to be (very) useful and only 6% consider it not useful [9]. Apart from digital health records, technology is expected to advance in various other ways over the next two decades, especially in the fields of minimally invasive technology, robotics, imaging, and diagnostics [10].

As the sustainable digital transformation of the healthcare system should also focus on creating health-promoting working environments for healthcare professionals, a question arises as to what connections exist between digitalization and ICT use and physicians’ burnout, work engagement and job satisfaction. In the following, the concept of technostress and the job demands–resources model (JD–R model), which form the basis for this research project, will be briefly described.

### 2.2. Theoretical Background and Literature Review

#### 2.2.1. Technostress

In the early 1980s, Craig Broad introduced the term technostress, implying that computer use was a possible cause of stress for users, based on his experience as a psychologist. He defined technostress as a “modern disease of adaption caused by an inability to cope with the new computer technologies in a healthy manner” and observed that this phenomenon was particularly prevalent among individuals who heavily relied on computers for their jobs [11]. The following factors contribute to technostress in the workplace: dependency on ICT [12], mismatch between technical requirements and user skills [13] and cultural work aspects such as multitasking, social isolation and distraction [14]. Following the transactional stress model, technostress is also described as the presence of technological environmental conditions that are assessed as demands or technostressors, which require individual adaptation as a coping mechanism [15].

The construct of technostress can be further differentiated into the following categories or technostress creators:

- Techno-overload: ICT forces employees to work faster and longer [16];
- Techno-complexity: the complexity of the ICT used in companies forces users to spend a lot of time learning new technical skills [17];
- Techno-insecurity: employees fear for their jobs and experience stress due to the possibility of being replaced by ICT or other employees with better technical skills [18];
- Techno-uncertainty: employees lose confidence in their technical skills and experience stress due to constant changes and updates or the use of new technologies [16];
- Techno-invasion (techno-omnipresence): ICT “invades” the private lives of users, as users are always available everywhere [13].

Moreover, so-called technostress inhibitors like literacy support provision, involvement facilitation and technical support provision act as antagonists to technostress [13]. Fourteen years after the original publication by Ragu-Nathan et al., the model has gained immense popularity and a variety of studies conducted in many industries are available on technostress [19]. For example, Ragu-Nathan et al. surveyed ICT end users from five different organizations and found that technostress creators decrease job satisfaction, leading to lower organizational and long-term commitment, while technostress inhibitors have the opposite effect. The authors also discovered that age, gender, education, and confidence in technology influence the intensity of technostress [13]. Further research has demonstrated a negative association between technostressors and job satisfaction [20] and identified techno-overload and techno-invasion as the “strongest” technostressors in this regard [21,22]. In contrast, a study by Hummert at al. showed that although the German tax consultants surveyed associated negative emotions with digitalization (e.g., fear of losing one’s job), they evaluated the real changes in their daily work so positively that their overall job satisfaction increased [23]. Technical and organizational support [24], employee involvement in the implementation phase and appropriate information management [25] were found to lower the incidence of technostress and promote employee well-being and productivity [26]. Studies of the relationship between technostress and employee health mainly focus on burnout [27,28]. The possible physiological symptoms of technostress include exhaustion [29], irritability and insomnia [30]. The psychological symptoms include frustration, increased psychological distress and time pressure [31] as well as feelings of ineffectiveness [29] and a reduction in job satisfaction, productivity, and work–life balance [15]. At this point, it should be noted that technostress differs in its manifestation and impact on employees’ working lives depending on the study setting and that results from studies conducted in other industries and countries cannot blindly be applied to the field of medicine without detailed consideration.

The Electronic Health Record (EHR) has been the focus of attention in research conducted on technostressors in medicine thus far [32,33,34,35], which is most likely explained by the fact that physicians use this technology for a variety of administrative tasks, including documentation, order entry, billing, and coding. For example, in a US study, primary care physicians spent an average of six hours per day working with the EHR, and an additional two hours were required for every hour of patient care [33]. The following factors related to EHR design have been identified as being associated with high stress levels and burnout: information overload, slow system response time, excessive data entry, difficulty navigating the system quickly, excessive note-taking, fear of missing important details, interference with the patient–clinician relationship, and notes prioritized for billing over patient care [32]. However, providing application training and optimizing workflows to be leaner and more effective have shown that using technology can reduce the time doctors spend in digital applications and allow for the delegation of responsibilities to other members of the patient care team [36]. In Finland, stress related to information systems was shown to increase during a nine-year period, especially for physicians in management positions. Changing, difficult, and poorly functioning information systems were identified as a significant source of stress [34]. Additionally, a study among Swiss psychiatric hospitals further highlighted that physicians and nurses experience the highest level of technostress among hospital staff and that technostress mainly occurs as a consequence of an imbalance between the effort and benefit of technology. In addition, technostress was found to be positively associated with burnout symptoms, headache and the intention to leave the profession or organization, and it was negatively associated with job satisfaction and general health status [37]. Since the number of digital applications implemented in German hospitals is low in international comparisons, there are currently few German study results concerning the use of ICT and specific outcomes for medical specialties [38], including technostressors in medical practice. No RCTs or meta-analyses about technostress specifically in the medical context are available to date [39]. With our study, we would therefore like to take stock of the degree of digitalization and the extent of technostress in German urological departments. This will help us to better understand the digital working environment of urologists and to better classify the results of this study in an international comparison.

#### 2.2.2. The Job Demands–Resources Model (JD–R Model)

The job demands–resources model (JD–R model) explains how job demands and job resources independently and interactively affect work-related experiences such as burnout and work engagement, and it served as a theoretical framework in our study. While job demands (e.g., physical workload, time pressure, personal customer interaction, challenging working environment, shift work) are defined as physical, psychological, social or organizational aspects of a job that lead to the individual having to exert more psychological or physical effort, job resources (e.g., constructive feedback, rewards, job control, participation, job security, supervisor support) are defined as working conditions that reduce job demands and related strain factors, stimulating personal growth and development and increasing work engagement [40]. The JD–R model can be used to predict occupational well-being and work motivation in any industry and company, and thus it forms the theoretical framework of many empirical studies [41].

Several studies have investigated job demands (e.g., high workload, time pressure, bureaucratic demands, work–family conflict) and resources (participation in decision making, development opportunities, leaders’ inspiration, relationships with colleagues or patients, meaning at work, patient recognition) specifically relevant to medical practice [42,43,44,45,46,47,48] and their effect on the outcomes burnout, job satisfaction, intention to leave to profession and quality of care. Due to the versatility of the JD–R model, many of the studies mentioned made specific modifications to the original model in terms of job demands, job resources, and the outcomes. However, so far there are no studies that combine the job demands–resources model with the construct of technostress to collect data on the outcomes physician burnout, job satisfaction and work engagement. Therefore, our study in particular aims to explore the digitalization-related support services available as job resources and coping mechanisms against technostress, to what extent they are used and how their benefits are evaluated. These findings could then serve as guidance for designing health-promoting work environments in hospitals, taking factors like technostress and its impact into account.

#### 2.2.3. Burnout, Work Engagement and Job Satisfaction in Urology

The WHO has formally defined burnout in the International Classification of Diseases (ICD-11) as a “syndrome conceptualized as resulting from chronic workplace stress that has not been successfully managed”, and it is characterized by the following three dimensions: “(1) feelings of energy depletion or exhaustion; (2) increased mental distance from one’s job, or feelings of negativism or cynicism related to one’s job; and (3) a sense of ineffectiveness and lack of accomplishment” [49].

Physician burnout is a growing concern in the modern era, with stressors such as increased non-direct patient care, clerical burden related to electronic medical records, longer working hours, and decreased work–life balance contributing to the problem [50]. US urologists have also described factors such as insurance and reimbursement, government regulations, and high medical and patient expectations as contributing to burnout [51]. In Germany, the estimated prevalence of burnout amongst physicians across all disciplines ranges from 4–20% [52]. However, urology has been identified as one of the worst specialties in terms of physician burnout and satisfaction with work–life balance in the US, with 63.6% of participating urologists reporting burnout compared to 41.2% in a 2011 study [53]. Another study found that trainees experience higher levels of depersonalization and lower levels of personal achievement, whereas urologists in the middle of their careers and those in private practice reported higher levels of emotional exhaustion [51]. Böhle et al. compared consultant urologists in a hospital and in private practice in a German cohort from Schleswig-Holstein and described increased levels of burnout on the subscales of emotional exhaustion and depersonalization among hospital urologists, urologists in training and urologists under 45 years of age [54]. European studies investigating burnout in urology have also presented comparable results in relation to this concerning trend [55]. However, the most recent representative German study dates back more than 20 years, which strongly indicates a need for further research in this specialty [54].

The consequences of physician burnout encompass poor work performance, medical mistakes, depression, substance abuse, difficulties in relationships with family and partners, and even suicidality. Proven strategies for prevention include resilience training, work–life balance through institutional support for protected time for personal activities (e.g., exercising, socializing, and reading non-medical literature), teamwork, and structured support programs like mentorship for residents [51,56,57]. According to meta-analyses, organizational interventions, such as modifying work processes, implementing shorter working hours and more breaks, open communication and feedback, supervisor support and mentoring, showed a significantly greater effect in terms of preventing physician burnout than individual interventions [58].

Work engagement is commonly defined as a positive, fulfilling, work-related state of mind that is characterized by vigor, dedication, and being absorbed in one’s field. This affective–motivational state can be seen as the antipode of occupational burnout, because it enables employees to be absorbed in their professional role and to contribute their energy and competences to their work in the best way possible [59]. According to a systematic review, physicians’ work engagement is high internationally [60]. This could be explained by a generally high experience of meaning in clinical work, which mediates the relationship between job demands and work engagement [61]. A study involving German surgeons showed that job resources in particular (influence at work, possibilities for development, degree of freedom at work, sense of community, feedback, quality of leadership, social support) are a strong predictor of work engagement [62]. Further studies also demonstrated an effect of work engagement on work ability and perceived quality of patient care [63,64].

Job satisfaction is commonly defined as a “pleasurable or positive emotional state resulting from the appraisal of one’s job or job experiences”. The major aspects of job satisfaction include work, pay, promotions, recognition, benefits, working conditions, supervision, co-workers, company and management [65]. Previous research concerning job satisfaction in urology is rather diverse. While one study described below-average values compared to other specialties as well as in international comparisons, another recent study reported high job satisfaction among urologists in Germany [66,67]. Younger and junior physicians generally seem to be more dissatisfied with their job, which might be influenced by variables such as working conditions, working hours, superiors, hierarchy, transparency, and participation in decision making [66,68]. Studies concerning the question of how the use of digital technology influences physician job satisfaction are inconclusive. It remains open whether digital technology and especially EHR use should be classified as a job demand or resource [69].

### 2.3. Objectives

Burnout, work engagement and job satisfaction are essential occupational variables in healthcare, with proven effects on general employee and patient satisfaction and health. In particular, staff absenteeism due to burnout and institutional turnover may be of special interest to hospital managers, not least in times of physician shortages. Our study has the purpose to fill the research gap in relation to the digital transformation, with the rapidly increasing implementation of ICT in inpatient care further highlighting the relevance of this study. We further aim to formulate recommendations for the implementation and handling of new technologies to reduce technostress and its negative effects on the working life of physicians.

**Figure 1:**
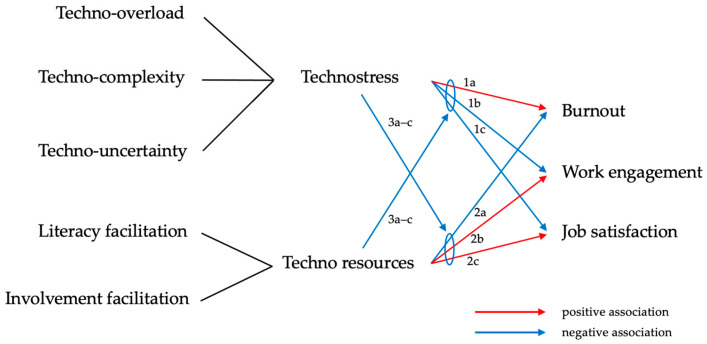
Conceptualized model of all the formulated hypotheses.

In our conceptual model, we followed the well-established original publication by Ragu-Nathan et al. and hypothesized the following: Technostressors (techno-overload, techno-complexity, and techno-uncertainty) are positively associated with (1a) burnout, and negatively associated with (1b) work engagement and (1c) job satisfaction. For techno-inhibitors (literacy facilitation and involvement facilitation), which according to Ragu-Nathan et al. act as counterparts to technostressors, we analogously assumed a negative association with (2a) burnout and a positive association with (2b) work engagement and (2c) job satisfaction [13]. Since, as mentioned above, job demands and job resources independently and interactively affect work-related experiences [40], we also hypothesized that technostressors and technostress inhibitors mutually moderate their associations with (3a) burnout, (3b) work engagement, and (3c) job satisfaction in the sense of a negative correlation. This third hypothesis is supported by the extended JD–R model, which proposes that “the interaction between work demands and work resources is also important for the development of workload and motivation” [40]. Work demands and work resources play a role here as moderating variables or buffers. This “buffer hypothesis” is consistent with further studies by other authors [70,71]. An overview of the conceptualized model of all the formulated hypotheses is provided in Figure 1.

The next section of this paper begins by describing the methods used to develop the survey and collect the data, followed by a step-by-step explanation of the statistical methods used in the data analysis. The results section begins by presenting the results regarding our conceptual model and continues with an analysis of the free-text responses. We conclude with a discussion of the results that focuses on the strengths, limitations, and practical implications of the study.

## 3. Materials and Methods

### 3.1. Study Design, Participant Selection and Data Collection

This study was conducted as a cross-sectional study using an online questionnaire in German. The survey consisted of checkbox questions as well as fields for free-text input, which was evaluated as part of the qualitative research. Participants were recruited in Germany and the data were collected between April 2022 and July 2022. Ethics approval was received from the Ethics Committee of the University Medical Center Hamburg-Eppendorf (Reference number LPEK-0423). Informed consent was obtained from all the subjects involved in the study. Participants were informed that the study was voluntary and that they could withdraw their consent at any time. The inclusion criteria for study participation were (1) working as a board-certified urologist or urology resident who has completed their first year of residency, (2) occupation in inpatient care, at least part time (≥20 h/week), and (3) daily professional use of ICT. These inclusion criteria were intended to ensure that the participating urologists had relevant long-term experience with ICT in their specialty. Furthermore, only departments with at least 5 employed urologists and an inpatient case number of at least 1000 per year were contacted to exclude outpatient clinics and visiting doctors from the study. A total of 299 urology departments meeting these criteria were contacted.

Recruitment was conducted in three different ways: E-mail contact with chief physicians, follow-up telephone calls with the secretaries of the departments, and direct e-mail contact with the practicing physicians in the clinical teams to further increase the number of participants. Contact information was obtained from the German Hospital Directory and online research [72].

### 3.2. Variables and Measurement

Based on our conceptual model and the JD–R model, technostress creators were assessed as job demands and technostress inhibitors as job resources. Both variables were examined as independent variables and moderators of the three outcome variables: burnout, work engagement and job satisfaction. The self-developed items were developed in German, as the data were collected in Germany. Table 1 provides an overview of the main variables and their measurement.

#### 3.2.1. Sociodemographic and Workplace Variables

Tools designed to assess age, gender, mother tongue, position, working hours in inpatient care, professional work experience, number of patient beds in the department, hospital ownership and federal state were developed by the study team.

#### 3.2.2. ICT Use and Perceived Usefulness

ICT use and perceived usefulness were treated as descriptive variables and assessed using internally developed as well as already established tools. The first internally developed assessment tool regarding ICT use was: “What types of information and communication technologies do you use for patient care in your daily clinical routine and how many hours of your working day are spent on average actively using these technologies?”, which was assessed by usage time and rated on a numerical scale of 0–10 h per day. As part of this assessment tool, participants evaluated the use of common technologies (hospital information system, electronic health records, patient portals, online knowledge databases, telemedicine, digital medication management, dictaphones, messenger services, tablets, virtual/augmented reality technologies, robotics, digital standard operating procedures/clinical pathways, decision support systems, smartphone apps). Furthermore, the perceived usefulness of ICT was measured using six items, PU1–6, from the Technology Acceptance Model (TAM) questionnaire on a five-point Likert scale (from 1 = strongly disagree to 5 = strongly agree). The exact wording was slightly adapted, e.g., “Using ICT would enhance my effectiveness on the job”, compared to the original publication [75]. Another internally developed tool assessed the availability of wi-fi and technical hardware (“How would you rate the availability of wi-fi and technical hardware (e.g., PCs, monitors, printers) at your workplaces?”), which was rated on a five-point Likert scale (1 = very good, 2 = good, 3 = satisfactory, 4 = sufficient, 5 = poor). Lastly, the participants were asked the question “Digitalization in everyday medical practice—a ‘curse’ or a ‘blessing’?” in an open-text field. This question was intended to further explore the participants’ subjective attitudes toward ICT qualitatively.

#### 3.2.3. Technostress Creators and Inhibitors

The items used to measure the technostress variables were adapted from Ragu-Nathan et al., who developed and empirically validated the Technostress Questionnaire [13]. The authors reported good reliability, with the Cronbach’s alpha values ranging from 0.77 to 0.87. Among the five dimensions of technostress, three were considered in the context of this study: techno-overload (five items), techno-complexity (six items), and techno-uncertainty (four items). The translated version was adapted from another German study of digital stress in organizations [76]. Techno-invasion and techno-insecurity were not included in the questionnaire due to the fact that doctors in the inpatient sector work shifts and usually do not have to worry about being replaced by colleagues with better technical skills. Of the three dimensions of technostress inhibitors, literacy facilitation, involvement facilitation and technical support provision, the latter was left out as there are usually no end-user helpdesks available in hospitals. All the items were presented on a five-point Likert scale (from 1 = strongly disagree to 5 = strongly agree).

#### 3.2.4. Burnout

For the assessment of burnout, the Copenhagen Burnout Inventory (CBI) was used. More specifically, six items on personal burnout were adapted and measured on a five-point Likert scale (from 1 = always to 5 = never/hardly ever). Studies showed satisfactory validity and high reliability (Cronbach’s alpha of 0.91 for the personal burnout scale) for the CBI instrument and its German version [77].

#### 3.2.5. Work Engagement and Job Satisfaction

The outcomes work engagement and job satisfaction were assessed using items from the well-validated and reliable German version of the Copenhagen Psychosocial Questionnaire (COPSOQ) [74]. For the German version, Cronbach’s alphas of 0.86 and 0.82 were reported for work engagement and job satisfaction [78]. The three items for work engagement (B14 1–3) and seven items for job satisfaction (B11 1–7) were presented on a five-point Likert scale (from 1 = very satisfied to 5 = highly unsatisfied resp. from 1 = always to 5 = never/hardly ever).

#### 3.2.6. Institutional Support Offers

The usage and perceived usefulness of institutional support offers were assessed through three internally developed questions. First, the participants were asked “To what extent do the following offers help you better deal with challenges in the context of ICT use in everyday life?” and then asked to rate various given options (individual trainings, group trainings, time management, communication training, IT hotline service, relaxation offers). They provided feedback regarding the availability of the support offer from their employer, their utilization of it, and their evaluation of its effectiveness, ranging from “very helpful” to “unhelpful”. The survey concluded with two additional free-text questions about the already available support offers or the lack thereof: “What other available offers help you to better deal with stress at work?” and “What other offers would you like to see from your employer?”.

### 3.3. Data Analysis

The analyses described below were conducted in a two-step process—first the quantitative data and then the qualitative data. Note that all the data were collected as part of the same survey. Responses received from 157 surveys were initially checked for plausibility, outliers, and missing values. The 116 completed datasets were the only ones which met the inclusion criteria and thus were included in the statistical analyses. To minimize the effect of internal dropouts, missing data were filled in based on multiple imputation, expecting data to be missing completely at random. Scales were built after reverse coding the outcome variables burnout, work engagement and job satisfaction. In the next step, descriptive analyses and Spearman correlations were performed to obtain a first overview of the dataset and the association between the variables (the results are presented under Section 4.2 and Section 4.3). In the following step, the Gauss–Markov assumptions were examined as prerequisites for the subsequently performed general model analysis and moderation analysis. To test for multicollinearity, the variance inflation factor was computed (VIF = 1.095), which is regarded as acceptable in cases where variables show values less than 3 [77]. After checking for normally distributed residuals, the assumption of heteroscedasticity was tested using the Breusch–Pagan test. This was met for multiple linear regressions. Therefore, further calculations were performed with the robust standard error HC3. The results of the general linear model and moderation analyses of the associations between technostress and the outcome variables burnout, work engagement and job satisfaction are presented below under Section 4.3.

Lastly, the answers from the free-text fields were analyzed in an inductive process according to the qualitative content analysis of Mayring [79]. Among the 116 included datasets, 97 participants also completed the free-text fields, which form the basis for our qualitative data analysis. In an iterative process, codes, categories, and sub-categories were identified and refined. The coding was reviewed reciprocally for accuracy and in consultation with the head of the research group. All the quotes used in the report (see Section 4.4 below) were translated from German to English by a person native in both languages [79]. All the statistical analyses were performed with IBM SPSS Statistics (version 27). The moderation analyses were also carried out using the PROCESS plugin for SPSS (version 4.1) by Andrew F. Hayes [80]. The answers from the free-text fields were analyzed in an inductive process according to the qualitative content analysis of Mayring [81]. We used MAXQDA 2020 (version 12) for the qualitative data analysis. To control for common method bias and increase the probability that respondents provided accurate answers to the questions, the following ex ante measures proposed by Podsakoff et al. were considered [82]. First, a good “cover story” was developed to decrease the tendency to respond in a socially desirable manner: Respondents were informed that data were obtained anonymously, how data will be used and how it might benefit them, and they were encouraged to provide truthful and non-influenced responses. Second, motivation was maintained by keeping the questionnaire short (median of 10 min), providing clear instructions (e.g., naming all the numerical scale points) and minimizing redundancies to the greatest extent possible. Third, positively and negatively worded items were balanced and the scale properties and response modes were varied to eliminate proximity effects in the questionnaire (e.g., checkbox questions, free-text fields, slide bars, different anchor labels). Since only standardized and well-validated items were used for the data collection of the main variables, no confirmatory factor analysis (CFA) was calculated.

## 4. Results

### 4.1. Sociodemographic and Occupational Characteristics of the Study Participants

The majority of the 116 participants were male (75.0%), senior physicians (50.0%) aged 30–49 years (56.0%) and working at least 50 h/week (41.4%) in public hospitals (58.6%). Further details concerning the sociodemographic and occupational characteristics of the participants are provided in Table 2.

### 4.2. Descriptive Statistics

The urologists reported particularly intensive use of the hospital information system (M = 5 h/day, SD = 2.27) and the electronic health records (M = 2.5, SD = 2.67), followed by dictaphones (M = 1, SD = 1.37) and patient portals (M = 1, SD = 1.92). An overview of this can be found in Table 3.

The wi-fi and hardware available in the department were rated on average as “satisfactory” on a scale from “very good” (1) to “poor” (5) (M = 2.88, SD = 1.02). The descriptive statistics of the main variables (technostress, burnout, work engagement and job satisfaction) can be found in Table 4. The variables technostress and technostress inhibitors were calculated using the mean values of their sub-dimensions. The Cronbach’s alpha (α) values ranged from 0.93 (for burnout) to 0.69 (for techno-uncertainty), higher than the recommended minimum value of 0.7 [83] with one exception, and can thus be considered satisfactory.

Among the institutional support offers presented to the 116 participants, IT hotline/support and ICT group trainings were most frequently offered by the employer (96.5% resp. 72.0%) and, if taken advantage of by the participants, were often rated as “helpful” or “very helpful” (37.0% resp. 16.6%). Also frequently offered by the employer but mostly not taken advantage of by the employee were relaxation offers (34.9%) and communication trainings (27.6%).

### 4.3. Associations between Technostress and the Outcomes Burnout, Work Engagement and Job Satisfaction

The Spearman correlation coefficients (ρ values) are highlighted in Table 5. Technostress was significantly correlated with burnout (positive association, ρ = 0.248), work engagement and job satisfaction (negative association, ρ = −0.176 and ρ = −0.170); however, the technostress inhibitors only showed a statistically significant correlation with job satisfaction (ρ = 0.203). While these values can be regarded as showing a weak to moderate association, the three outcome variables burnout, work engagement and job satisfaction were correlated strongly with each other [84].

The general linear models indicate that the independent variables predicted the outcome burnout most highly (R^2^ = 0.073, *p* < 0.001), followed by job satisfaction (R^2^ = 0.062, *p* < 0.001) and work engagement (R^2^ = 0.042, *p* < 0.001). In all the models, technostress was significantly associated with burnout, work engagement and job satisfaction. The highest association was found for burnout, with an increase of 0.293 (*p* < 0.001) associated with an increase in technostress of 1 point in a possible range of 1 to 5. Technostress was also negatively associated with job satisfaction (β = −0.206, *p* < 0.001) and work engagement (β = −0.175, *p* < 0.001). Therefore, the 1a–c hypotheses can be confirmed. However, the effects of the independent variable technostress inhibitors were only significant on the outcome variable job satisfaction in the sense of a positive association (β = 0.119, *p* < 0.001). So, while hypothesis 2c could be confirmed, hypotheses 2a and 2b had to be rejected due to the lack of significance, indicating that there is no significant relationship between the degree of techno resources, burnout, and work engagement. The results are presented in Table 6.

Furthermore, a moderation analysis was performed to determine whether the interaction between technostress and the technostress inhibitors significantly predicted burnout, work engagement and job satisfaction. The overall model was significant for the outcomes job satisfaction (*p* < 0.001), predicting 7.94% of the variance, and work engagement (*p* < 0.001), predicting 4.82% of the variance, but not for burnout. While the moderating effect on burnout assumed in hypothesis 3a therefore had to be rejected, the moderating role between technostressors and technostress inhibitors hypothesized in 3b and 3c was confirmed. The results of the moderation analysis can be found in Table 7.

### 4.4. Free-Text Answer Analysis about (Dis)Advantages of ICT and Institutional Support Offers

In the following, the results of the free-text answers analysis are summarized into categories and sub-categories, which are presented in tables and example quotes from the participants. The number of mentions of the subcategories is given in parentheses after each category (*n* = x). In total, 97 urologists answered the qualitative study questions. A total of 93 out of 116 participants described numerous advantages and disadvantages related to ICT use (results see Table 8).

On the one hand, the advantage of having patient data available quickly and at any time in several places at the same time was reported particularly frequently (*n* = 26).

*“You have almost all the medical reports and info available on any PC anywhere in the hospital 24/7.”*

On the other hand, however, there was a perceived increase in the time required for ICT use (*n* = 19).

*“Prescribing medication and requesting nursing services, as well as documenting patient care takes about 3x as long as on paper.”*

In addition, various technical problems concerning hardware and wi-fi (*n* = 37), user-friendliness (*n* = 15) and lack of interoperability (*n* = 8) were reported and criticism of the digitalization strategy of the clinics was expressed (*n* = 19).

*“When implemented correctly, digitization is a blessing and definitely the future. For us, this blessing is mainly called crashes and waiting times (hourglass) and is therefore more of a curse. If the software and hardware are not powerful enough, digitization is a step backwards in efficiency and regularly causes frustration.”*

*“Things are moving too slowly. We need better digital applications and interconnections between sectors fast.”*

The answers to the second question about the already available support offers to better deal with stress at work, which was answered by 45 out of 116 participants, can be summarized as institutional offers, organizational changes, and other factors (results see Table 9).

Among the helpful institutional offers, relaxation offers such as break rooms (*n* = 1), massage or physiotherapy (*n* = 3) and sport (*n* = 4) were named most frequently, but with the comment that participation in these offers was not possible or not welcomed due to the high workload of the doctors.

*“There are no offers for stress reduction, it was not allowed to actually take part in the in-house exercise training during breaks.”*

*“1 × 15 min shoulder massage in physiotherapy due to the stress caused by the Corona pandemic; also, corporate benefits for visiting a public indoor swimming pool”*

In addition, desired organizational changes such as individual working hours or a reduction of working hours (*n* = 6) as well as better clinical process management (*n* = 3) were mentioned.

*“Better hospital management, better resource management, less influence by hospital economists.”*

*“More competent staff in IT so that improvements are actually implemented.”*

For many participants, private leisure time activities were also an important way to relieve stress (11/116).

*“I do not take advantage of ‘relief opportunities’ that may be offered by the employer. Partnership, friendships, spirituality, nature and exercise are essential for me.”*

A total of 56 urologists answered the third and final free-text question about the desired support offers that would help them better manage stress at work, if available. Again, their answers were categorized into institutional offers, organizational changes, and other factors (results see Table 10).

Besides relaxation (*n* = 5) and sports offers (*n* = 7), communication training, e.g., systemic coaching, practicing non-hierarchical forms of communication, leadership training, Balint groups (*n* = 4) and individualized workplace design (*n* = 3) were most frequently described by the respondents.

*“Adequate workplace equipment, back-friendly seating/desk chairs, height-adjustable desk, functioning IT that is also practicable and does not constantly hang up or take forever to load; providing a second monitor etc.”*

Regarding organizational changes, a desire for more staff and better work distribution (*n* = 14) as well as for the involvement of medical end users in digitization projects (*n* = 5) were frequently expressed.

*“The main problem is the lack of staff. For us, it’s more in the nursing environment. If there was more time to explain things, some things would be easier. I am much more stressed because I always must ‘iron out’ frustration and inexperience etc. among the nursing staff and residents.”*

*“The offer that administrative tasks coding, discharge management, surgery planning, bed planning) are taken on by other persons (physician assistant). In terms of responsibilities, more and more tasks are labelled ‘medical’ activities. This leaves little time for practicing medicine. This is compensated by working faster. We therefore no longer speak of overtime, but of ‘work density’/hour.”*

*“More real involvement in the development of (IT) processes that massively influence the workflow. Especially in IT, minimal changes can only ever be enforced by the group of end users (doctors, nursing) at the end of the implementation processes. Here, an earlier involvement of the users in the decision-making processes must take place.”*

## 5. Discussion

This study contributes to the emerging topic of technostress among physicians in an inpatient care setting. It provides first insights into the association of technostress with burnout, work engagement and job satisfaction in urology. Furthermore, the usage and perceived usefulness of ICT in urology are determined for the first time across Germany and recommendations to reduce and better manage technostress for urologists working in inpatient care are formulated (see Section 5.3).

### 5.1. Key Results

While the current literature on digitalization and ICT in healthcare shows many convincing economic and patient-related benefits, contradictory results are emerging for the effect it has on clinicians [85,86]. In our study, technostress was overall moderately pronounced among the participants, similar to existing research involving healthcare workers from Germany and Switzerland [37,87]. While techno-overload was found to be comparable to the original publication by Ragu-Nathan et al., the scores for techno-complexity and techno-uncertainty in this study were lower than those seen in other industries [13]. This makes sense, as digital applications in hospitals are rather documentation- and billing-oriented. ICT in healthcare is therefore generally less complex and developing less rapidly compared to other sectors. A meta-analysis of technostress studies from various sectors consistently showed the negative association of technostress with job satisfaction and an increase in burnout [88]. This also applies to the urologists participating in this study, as a low to moderate but significant negative association of technostress with burnout and a positive association with job satisfaction and work engagement could be confirmed. This could be explained by the doctor’s triple burden, including teaching, research, and clinical practice, which is exacerbated by technostress and interruptions to patient care through ICT. However, the results in the literature regarding this topic are inconsistent, and it is debatable whether employees could even perceive technostress as an opportunity or challenge that might increase their work motivation [89].

This study found low scores among both surveyed techno inhibitors, literacy facilitation and involvement facilitation. This was especially true for involvement facilitation and its association with the outcomes burnout, work engagement and job satisfaction, which was either not significant or very low. One possible reason for this might be that increased participation in IT processes might be perceived of as an additional burden and responsibility. Furthermore, it is unclear whether involvement and literacy facilitation are relevant factors or whether and which other techno resources, such as appropriate information management [25], should be included in further analyses.

Fortunately, our study also did not reproduce the alarming burnout rates of over 60% seen among US urologists [53]. The participants in our study reported a low to moderate level of burnout according to the cut-off levels of the CBI scale. However, it should also be noted that this study used a different instrument, the Copenhagen Burnout Inventory, instead of the Maslach Burnout Inventory. Compared to German urologists, US physicians report higher working hours, increasing computerization of practice and decreasing reimbursements more often as factors responsible for causing burnout [90]. Physicians internationally reported too many bureaucratic tasks (e.g., charting, paperwork) as the “Top Burnout Factor” [73], which further highlights the relevance and potential of digitalization in automizing administrative and documentation duties and thus improving working conditions in hospitals.

In summary, technostress does not seem to be an acute issue for the urologists who participated in this study. However, we encourage the monitoring of future developments along with burnout, work engagement and job satisfaction under the aspect of the rise and implementation of new digital technologies. Furthermore, as techno-stressors can quickly become outdated, repeated conceptual work is required to include new phenomena. Although the advantages of digitalization in healthcare organizations are promising and crucial, new technology must be evaluated critically before being implemented. When discussing the relevance of technostress, it should also be mentioned that other studies showed further associations with the long-term consequences for healthcare workers, such as headache, mental and physical health, high cortisol levels and intention to leave the organization or the profession [37,91]. In addition, other aspects of technostress that were not considered in this study seem to be relevant for healthcare workers, such as work–private life conflicts and the quantitative demands at work, which are only partially incorporated into the technostress scale [92,93,94].

The qualitative analysis of the free-text responses extends and complements these and previous findings. While technical problems, low user-friendliness, lack of interoperability, documentation burden, unsatisfactory training offers and missing involvement in strategic digitalization decisions are perceived as “curses” of ICT use, the availability of patient information and increased transparency are assessed positively. It is striking that some participants reported that the use of ICT saves time, while others reported the complete opposite. Cohen et al. described this phenomenon as the “Empowerment/Enslavement Paradox” in their study concerning the use of personal communication devices among surgeons [95]. In summary, the results reflect the ambivalences of the digital transformation and the perceived imbalance between effort and outcome. In a recent survey among German urologists, 74% said they considered the digitalization process reasonable and 34% had partial or strong concerns about the complete digitalization of patient documentation. The study further concluded that German urologists, and especially the younger age group, are open to the digitalization process and implementation of ePA [96]. The primary problem thus does not seem to be a lack of motivation but rather a lack of “smart”, user-friendly, and practical digital solutions for the inpatient care setting. Other authors have already described different kinds of organizational barriers that partly prevented hospitals from improving the digital work environment more efficiently, such as long-lasting time lags in accomplishing changes, judicial regulations, difficulties in changing work routines, and budget restrictions [22].

### 5.2. Strengths and Limitations

The research-related contribution of this paper lies in two domains. In terms of ICT research, this study provides empirical validation of the construct of technostress and investigates its relationships with individual outcomes like burnout, work engagement and job satisfaction for urologists working in an inpatient care setting in Germany. In the organizational behavior domain, this study adds to the transaction-based approach by identifying stressors associated with the use of ICT and evaluating institutional support offers as coping mechanisms [97].

However, this study had several limitations. First, only 25% of participants were female, which corresponds to the approximate gender distribution in the field. In addition, the group sampled did not reflect the actual distribution of urologists found in a hospital setting, as chief and senior physicians were overrepresented (66.4%) compared to their usual representation at 14.8% [98]. This can be partly explained by the recruitment strategy via chief physicians and their secretaries, and partly by a possibly greater interest in the study on the part of senior urologists with management responsibilities and in leadership positions. In addition, a response rate of 21.6% (116 completed surveys out of 538 surveys) started is considered low but rather common for web-based surveys among health professionals [37,54,99]. Therefore, the results must be interpreted with caution due to the small sample size of this study, especially considering junior and female doctors.

The following factors could partially explain the low response rate in this case:

- Since urology is an operative specialty, their main workplace is the operating room rather than at a PC in an office.
- E-mails often do not get through spam filters due to increased security measures.
- In addition to the already high workload among physicians, staff shortages have become even more acute as a result of the COVID-19 pandemic in 2022. Thus, it only seems reasonable to prioritize patient care tasks above participation in an online survey.
- E-mail is not necessarily the main communication channel in many clinical teams and therefore e-mails may not be read.
- Chief physicians and secretaries did not forward the survey link, either due to lack of interest in the topic or to avoid further burdening the physicians in their team.

In addition, even though the questionnaire we developed can be applied to other medical specialties, the results of this study should not be transferred to other specialties without further consideration. Moreover, no causal conclusions can be drawn, as this study used cross-sectional data. These implications must all be considered when interpreting our results.

### 5.3. Implications for Research

The digital transformation of German hospitals will progress rapidly over the next few years, not least as a result of the Hospital Future Act. This highlights the need for further studies like ours, discussing health-promoting working environments for healthcare professionals in the context of digitalization. We encourage future researchers to conduct interdisciplinary studies, e.g., in different medical specialties or in outpatient care, as well as longitudinal studies to better understand and monitor the developments and effects over the course of the digital transformation. Interventional studies in the context of implementing new technologies or introducing support offers could further point out and explain the direct effects on work processes and employee health outcomes. Given the generally low healthcare worker response rates to online surveys, a question arises as to how and through which communication channels medical staff could be effectively recruited for future research projects.

### 5.4. Practical Implications

Based on the feedback from the physicians participating in our study, we formulated the following eight recommendations, which are divided into organizational and behavioral aspects, designed to reduce and better manage technostress for urologists working in inpatient care.

Firstly, offering a wide range of corporate benefits can provide a balance to the high demands of daily clinical routine and make clinics an attractive employer for medical staff and potential applicants. Examples of possible benefits are relaxation offers (break rooms, massages, physiotherapy), sports offers (exercise during breaks, sports courses like yoga or running, company bicycles, discounts for local gyms or swimming pools), childcare, communication trainings, healthy/free food offers in the cafeteria or break rooms, individualized workplace designs (with height-adjustable desks or ergonomic desk chairs), overtime payment and individualized working hours. In addition, providing modern hardware and software with a focus on user-friendliness (fast system response time, intuitive navigation, appealing user interface, alignment to clinical processes) as well as fast wi-fi guarantees smoother clinical workflows with less technical disruptions and failures and higher end-user satisfaction. In addition, physicians should be provided with reliable IT support, with good availability and response time to report and solve technical problems quickly. Moreover, recruiting more medical and nursing staff and distributing tasks between physicians and medical support staff to help with coping should the workload and work density intensify through the digital transformation (digital documentation and administration like coding, digital discharge management, and bed planning).

On the behavioral level, making use of ICT training through more (personally) structured ICT trainings or ICT trainings in small groups is essential for the confident and professional handling of ICT in the clinical routine. In order to digitalize hospitals in a demands- and needs-oriented manner, physicians should also be involved in the planning and implementation phases of digitization projects as end users. Furthermore, promoting good communication and a positive work atmosphere in clinical teams makes physicians feel more appreciated for their work and encourages teamwork and sharing knowledge in dealing with new technologies. Lastly, awareness of technostress and its effects at the leadership level, as well as active exchange on new technical innovations and stress reduction, e.g., in the context of conferences or shadowing, can inspire and facilitate individual change management in hospital digitization.

## 6. Conclusions

To the best of our knowledge, this study was the first quantitative study to apply the JD–R model to the context of urologists working in an inpatient care setting in Germany [40]. This study also complements the technostress literature contributing to theory and practice [13,15]. The results provide new information on ICT use and support offers, technostress and its relationships with physician burnout, work engagement and job satisfaction in urology. Further research is needed to better understand and monitor the developments and impacts of the digital transformation in medicine on healthcare professionals.

## Figures and Tables

**Table 1:** List of main independent (IV) and dependent (DV) variables and measurement.

Construct (Type of Variable)	Measurement and Source	No. of Items
Technostress creators (IV, moderator)	Technostress Questionnaire [13]	14
Technostress inhibitors (IV, moderator)	Technostress Questionnaire [13]	9
Burnout (DV)	Copenhagen Burnout Inventory [73]	6
Work engagement (DV)	COPSOQ [74]	3
Job satisfaction (DV)	COPSOQ [61]	7

**Table 2:** Descriptive statistics of the sample.

Variable	*n*	%	Variable	*n*	%
Gender			Patient beds in the department		
Female	29	25.0	10–19	2	1.7
Male	87	75.0	20–29	40	34.5
Age			30–39	39	33.6
20–29	11	9.5	40–49	20	17.2
30–39	35	30.2	50 and more	15	13.0
40–49	30	25.9	Hospital ownership		
50–59	24	20.7	Private	26	22.4
60 years and older	16	13.8	Public	22	19.0
Mother tongue			Private non-profit	68	58.6
German	106	91.4	Federal state		
Other	10	8.6	Baden-Württemberg	17	14.7
Position			Bavaria	6	5.2
Chief physician	19	16.4	Berlin	6	5.2
Senior physician	58	50.0	Brandenburg	5	4.3
Attending physician	16	13.8	Bremen	3	2.6
Resident physician	23	19.8	Hamburg	7	6.0
Years of professional work experience			Hesse	13	11.2
1–9	36	31.0	Mecklenburg Western Pomerania	5	4.3
10–19	31	26.7	Lower Saxony	13	11.2
20–29	25	21.6	North Rhine-Westphalia	35	21.6
30–39	21	18.1	Rhineland Platinate	2	1.7
40 years and more	4	3.4	Saarland	0	0.0
Working hours in inpatient care/week			Saxony	2	1.7
20–29	11	9.5	Saxony-Anhalt	4	3.4
30–39	13	11.2	Schleswig Holstein	7	6.0
40–49	42	36.2	Thuringia	1	0.9
50 h and more	48	41.4			

**Table 3:** ICT usage time in hours per day.

	HIS	EHR	Dictaphones	Patient Portals	Robots	Medication Management	Smartphone Apps	Online Knowledge Bases	Digital SOPs	Tele- Medicine	Messenger Services	Tablets	Decision Support Systems	VR/AR
M	5.000	2.422	0.940	0.914	0.884	0.806	0.685	0.681	0.491	0.461	0.375	0.306	0.108	0.017
Mdn.	5.000	1.500	0.500	0.000	0.000	0.000	0.500	0.500	0.250	0.000	0.000	0.000	0.000	0.000
SD	2.2660	2.6683	1.3676	1.9261	1.6102	1.6950	1.2204	1.1542	0.9041	0.8292	1.0792	1.2437	0.3217	0.1857
Min	1.0	0	0	0	0	0	0	0	0	0	0	0	0	0
Max	10.0	10.0	7.5	10.0	6.0	10.0	10.0	8.5	8.0	5.5	10.0	10.0	2.0	2.0

**Table 4:** Descriptive statistics of the main variables.

Variables	M	Mdn.	SD	Min	Max	α
Technostress	2.67	2.71	0.692	1	5	
Techno-uncertainty	2.82	2.75	0.776	1	5	0.690
Techno-complexity	2.25	2.00	1.040	1	5	0.867
Techno-overload	2.99	3.10	0.945	1	5	0.853
Technostress inhibitors	2.30	2.17	0.818	1	5	
Literacy facilitation	2.64	2.60	0.887	1	5	0.846
Involvement facilitation	1.97	1.75	0.920	1	5	0.837
Job satisfaction	3.56	3.57	0.591	2	5	0.793
Work engagement	3.73	3.67	0.671	2	5	0.790
Burnout	2.86	2.92	0.788	1	5	0.907

**Table 5:** Spearman correlation analyses.

Variables	1	2	3	4	5
1 Technostress Techno-overload Techno-complexity Techno-uncertainty	–				
2 Technostress inhibitors Literacy facilitation Involvement facilitation	0.234 **				
3 Burnout	0.248 **	0.040	–		
	0.245 **	0.063 *			
	0.181 **	−0.003			
	0.201 **				
4 Work engagement	−0.176 **	0.020	−0.441 **	–	
	−0.149 **	0.012			
	−0.242 **	−0.031			
	−0.094 **				
5 Job satisfaction	−0.170 **	0.203 **	−0.560 **	0.437 *	–
	−0.139 **	0.198 **			
	−0.184 **	0.118 **			
	−0.111 **				

** correlation is significant at less than 0.01 (2-tailed); * correlation is significant at 0.05.

**Table 6:** Results of the general linear model (GLM) analysis.

Variable	Burnout		Work Engagement		Job Satisfaction	
	R^2^ = 0.073 F_2,1273_ = 50.231, *p* < 0.001		R^2^ = 0.042 F_2,1272_ = 28.137, *p* < 0.001		R^2^ = 0.062 F_2,1273_ = 42.218, *p* < 0.001	
	β	robust se	β	robust se	β	robust se
Technostress	0.293 *p* < 0.001	0.029	−0.175 *p* < 0.001	0.023	−0.206 *p* < 0.001	0.022
Technostress inhibitors	0.036 *p* = 0.189	0.028	−0.049 *p* = 0.082	0.028	0.119 *p* < 0.001	0.030
Intercept	1.991 *p* < 0.001	0.091	4.308 *p* < 0.001	0.080	3.839 *p* < 0.001	0.081

β = estimated beta value; robust se = robust standard error.

**Table 7:** Results of the moderation analysis.

Variable	Burnout		Work Engagement		Job Satisfaction	
	R^2^ = 0.073 F_2,1272_ = 40.046, *p* < 0.001		R^2^ = 0.048 F_3,1271_ = 23.899, *p* < 0.001		R^2^ = 0.079 F_3,1272_ = 36.415, *p* < 0.001	
	β	robust se	β	robust se	β	robust se
Moderation effect	−0.020 *p* < 0.001	0.462	−0.081 *p* = 0.001	0.030	−0.123 *p* = 0.001	0.031

β = estimated beta value; robust se = robust standard error.

**Table 8:** Advantages and disadvantages of ICT use.

Disadvantages/Criticisms of ICT
Technical problems: lack of hardware/outdated hardware (*n* = 9), slow wi-fi (*n* = 3), technical failures/difficulties (*n* = 12), slow system response time (*n* = 11)
Low user-friendliness: complicated navigation (*n* = 3), missing coordination with clinical work processes (*n* = 5), lack of user-friendliness (*n* = 5), complexity of ICT (*n* = 1), lack of user motivation (*n* = 1)
Lack of interoperability (*n* = 8)
Clinical documentation: high documentation load (*n* = 6), double documentation (*n* = 3)
Increased workload (*n* = 19)
IT support and training: lack of IT support (*n* = 2), lack of training offers (*n* = 2)
Digital transformation in the hospital: delayed transformation process (*n* = 11), additional work and problems due to transition phase (*n* = 8)
**Advantages of ICT**
Availability of information: availability of patient data quickly and at any time in several places at the same time (*n* = 26), use of online knowledge databases (*n* = 1)
General reduction of workload and time savings (*n* = 10)
Transparency: transparent documentation (*n* = 3), better overview and readability (*n* = 2)
Desire for paperless work and digital transformation (*n* = 3)

**Table 9:** Available support offers to better deal with stress at work.

Category	Sub-Categories
Institutional offer	Break room (*n* = 1), massage/physiotherapy (*n* = 3), sports facilities (*n* = 4), childcare (*n* = 1), communication training (*n* = 1), higher salary (*n* = 1)
Organizational changes	Better process management (*n* = 3), expansion of IT support (*n* = 2), individual working hours/reduction of working hours (*n* = 6), working time accounts (*n* = 1), good team atmosphere (*n* = 2), good communication within the team (*n* = 2)
Other	Private recreation (*n* = 8), none (*n* = 11)

**Table 10:** Non-available support offers to better deal with stress at work.

Category	Sub-Categories
Institutional offer	Relaxation offers (*n* = 5), sport offers (*n* = 7), healthy/free food (*n* = 2), communication training (*n* = 4), individualized workplace design (*n* = 3), childcare (*n* = 1), higher salary or payment of overtime (*n* = 2)
Organizational changes	More staff, needs-based demand planning work distribution (*n* = 14), more ICT training offers (*n* = 2), personal ICT training (*n* = 2), expansion of IT support (*n* = 6), involving end-users in digitization projects (*n* = 5), more appreciation (*n* = 2), individual working hours or reduction of working hours (*n* = 5), adherence working hours according to the employment contract (*n* = 1), working time account (*n* = 1)
Other	User-friendly ICT in general (*n* = 1), better hardware (*n* = 1), faster Wi-Fi (*n* = 1), interoperability of ICT (*n* = 2), shorter loading times (*n* = 1), none (*n* = 4)

## Data Availability

The data in this study are not publicly available due to German national data protection regulations.
